# *In vitro* Effects of Albendazole on *Raillietina echinobothrida*, the Cestode of Chicken, *Gallus domesticus*

**DOI:** 10.4103/0975-1483.71630

**Published:** 2010

**Authors:** K Lalchhandama

**Affiliations:** *Department of Zoology, Pachhunga University College, Mizoram University, Aizawl - 796 001, India*

**Keywords:** Albendazole, microtriches, *Raillietina echinobothrida*, scanning electron microscopy, tegument

## Abstract

Albendazole, a member of benzimidazole group of compounds, has been shown to have a broad spectrum activity against all classes of helminth parasites. Although it has also been experimentally proven to be effective against cestode infection of poultry, the actual effects of the drug are not yet described. The present in vitro study demonstrated that the commercial prescription drug Zentel® was significantly effective against adult *Raillietina echinobothrida* Mégnin, the major cestode parasite of domestic chicken, *Gallus domesticus* Linnaeus. It clearly exhibited dose-dependent lethal activity at the different concentrations that were tested. Scanning electron microscopy (SEM) revealed that the drug caused extensive structural alterations on the body surface of the cestode. Severe contraction and shrinkage were evident throughout the entire length of the body. The suckers on the scolex became invaginated due to shrinkage. The distinct body segments, the proglottides, were completely distorted. The fine hairy microtriches on the tegument were obliterated and in its place were formed abnormal clumps of tissues. The results of this investigation are in favor of the use of albendazole as a drug of choice in the management of poultry helminthiasis.

## INTRODUCTION

Poultry husbandry is by far the most common in the livestock sector throughout the world. There is an increasing global demand for poultry meat and eggs as a consequence of the ever increasing human population. Rapid growth in the demand for livestock products is being met by a corresponding growth in the poultry industry. In India, poultry meat and egg production has been the fastest growing in agricultural or livestock production, with an average growth of 8% per annum.[1] But with increase in poultry management, a range of different parasitic infections are re-emerging, which are a serious hindrance to successful economic output.

The tapeworms belonging to the genus *Raillietina* are the most prevalent avian helminth parasites throughout the world. *R. echinobothrida* Mégnin, 1880, is the most important species in terms of prevalence and pathogenicity, particularly in the domestic fowl, *Gallus domesticus* Linnaeus, 1758.[2] The cestode inhabits the small intestine and causes stunted growth of young chicken, emaciation of the adult, and decreased egg production of the hen.[3] In conditions of heavy infestation, *R. echinobothrida* is listed as one of the most pathogenic tapeworms, causing conspicuous intestinal nodules in chicken, with characteristic hyperplastic enteritis associated with the formation of granuloma.[4] The symptom is termed ’nodular tapeworm disease’ in poultry. Intestinal nodules often result in degeneration and necrosis of intestinal villi and ultimately lead to death. Unfortunately, there are no prescription drugs for the treatment of avian cestodes. Anthelmintic intervention often involves medication with piperazine, tetramisole, and oxfendazole. However, these anthelmintics generally exhibit low efficacy and are associated with undesirable side effects.[5]

Albendazole (methyl [6-(propylthio)-*1H*-benzoimidazol-2-yl]carbamate (C_12_H_15_N_3_O_2_S) is a member of the benzimidazole group of compounds and was discovered to be a broad spectrum anthelmintic, effective against all classes of helminth parasites.[6] Due to its high potency and mild side effects it has become the drug of choice against most human and veterinary helminths.[7,8] Albendazole has been reported to be highly effective against poultry cestodes. It showed 100% efficacy against *Raillietina tetragona* infection in experimentally infected layer chicken.[9] It was also effective in deworming chickens infected with *R. cesticillus* (96.2% reduction) and caused no adverse effects on the host.[10] However, the nature of its effect on the cestodes are not yet investigated. The present investigation, therefore, is an attempt to assess the efficacy of albendazole and its effects on the structural characteristics of *R. echinobothrida*, the most important cestode of poultry.

## MATERIALS AND METHODS

### Recovery and *in vitro* treatments of cestodes

Native live fowls (*G. domesticus*) were obtained from the poultry abattoir at Aizawl, India. Prior approval of the ethics committee was obtained and the animals were handled in accordance with the guidelines of CPCSEA. They were sacrificed at the Department of Zoology, Pachhunga University College, Aizawl, and on immediate autopsy, live adult *R. echinobothrida* were recovered from the intestines. The worms were collected in 0.9% neutral phosphate-buffered saline (PBS, pH 7–7.3) and then incubated at 37 ± 1°C in a glass-chambered automated incubator. Different concentrations, viz, 0.5, 1, 2, 5, 10, and 20 mg/mL, of albendazole were prepared by dissolving in PBS supplemented with 1% dimethylsulfoxide (DMSO). A batch of five cestodes was introduced into each concentration. One group of worms was maintained as control in a medium containing only PBS with 1% DMSO. Each experimental assay consisted of five replicates. Motility and mortality of the worms were observed, and the time taken for paralysis and death was recorded. Paralysis was defined as complete loss of spontaneous motor activity upon physical stimulation of the worms. Dipping the parasites in tepid PBS (~45°C) induced movement in sentient worms; if no movement occurred upon such stimulation, death was confirmed.

### Scanning electron microscopy

Cestodes were selected from the control and the 20 mg/mL albendazole-treated groups. They were fixed in 10% neutral cold-buffered formaldehyde at 4°C for 24 h. After fixation in 1% buffered osmium tetraoxide for 1 h, they were washed with PBS and then dehydrated through ascending concentrations of acetone up to pure acetone. They were then treated with tetramethylsilane for 10 min and then air-dried under room temperature. After coating with gold in a fine-coat ion sputter, JFC-1100 (JEOL), and mounting on metal stubs, the specimens were observed using a LEO 435 VP scanning electron microscope (SEM) at an electron accelerating voltage of 20 kV.

### Chemicals and drug

Except where otherwise stated, all the chemicals and reagents used were of standard analytical grade, obtained either from Merck or S.D. Fine Chemicals Limited, India. Methanol was procured from Qualigens, India, and albendazole (Zentel®) was a product of GlaxoSmithKline Pharmaceutical Limited, Mumbai, India.

### Data analysis

All data are presented as means ± the standard deviation (SD) of the mean. Comparison of the mean values between the treated and control groups was made using unpaired Student’s *t*-test, and the probability value was considered significant at *P*<0.05.

## Results

### Motility and survival effects

Observations on the *in vitro* efficacy of albendazole in terms of motility and mortality of *R. echinobothrida* are shown in Table 1. The results indicate that the drug exhibited dose-dependent paralytic and lethal effects on the cestode. The worms maintained in the control medium containing only PBS with DMSO survived very well up to 54.78 ± 0.7 h. Treatment of the cestodes with 0.5, 1, 2, 5, 10, and 20 mg/mL of albendazole resulted in paralysis at 17.07 ± 0.6 h, 12.94 ± 0.6 h, 9.62 ± 0.6 h, 3.40 ± 0.4 h, 1.32 ± 0.2 h, and 1.12 ± 0.3 h, respectively; complete loss of life occurred at 27.10 ± 0.7 h, 18.25 ± 0.4 h, 12.98 ± 0.5 h, 5.72 ± 0.4 h, 3.22 ± 0.3 h, and 1.85 ± 0.4 h, respectively. Thus, albendazole was evidently a potent anthelmintic against the poultry cestode and possessed significant dose-dependent efficacy at all concentrations tested.

**Table 1:** Dose-dependent efficacy of albendazole on the viability of *R. echinobothrida*

Test group	Dose mg/mL	Time taken (in hours) for	
		Paralysis	Death
Control	0		54.78 ± 0.7*
Albendazole	0.5	17.07 ± 0.6	27.10 ± 0.7*
	1	12.94 ± 0.6	18.25 ± 0.4*
	2	9.62 ± 0.6	12.98 ± 0.5*
	5	3.40 ± 0.4	5.72 ± 0.4*
	10	1.32 ± 0.2	3.22 ± 0.3*
	20	1.12 ± 0.3	1.85 ± 0.4*

Values are expressed as mean ± SD (*n* = 5);

* Student’s *t*-test; significantly different at *P*<.05 as compared to control

### Morphological structure and changes in the cestode

**Figure 1:**
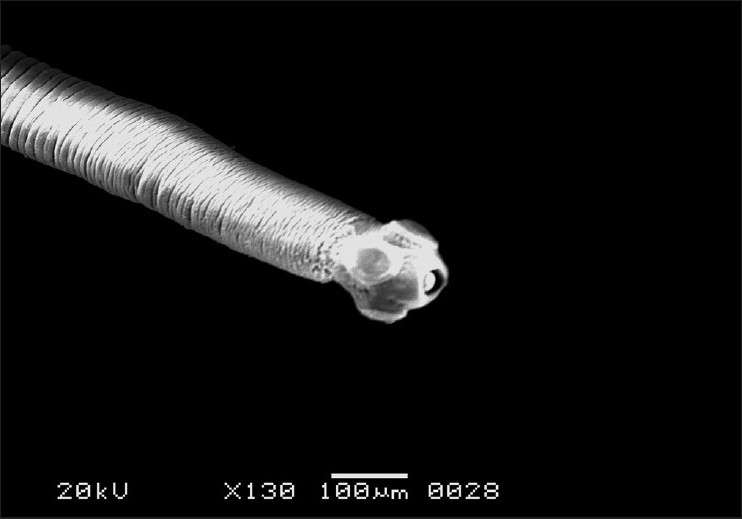
Scanning electron micrograph of untreated *R. echinobothrida* showing the anterior part of the body. The extreme terminal end is the scolex bearing holdfast organs such as the four semicircular suckers and the rostellum. The scolex is followed by a short neck and then a series of ribbon-like proglottides

**Figure 2:**
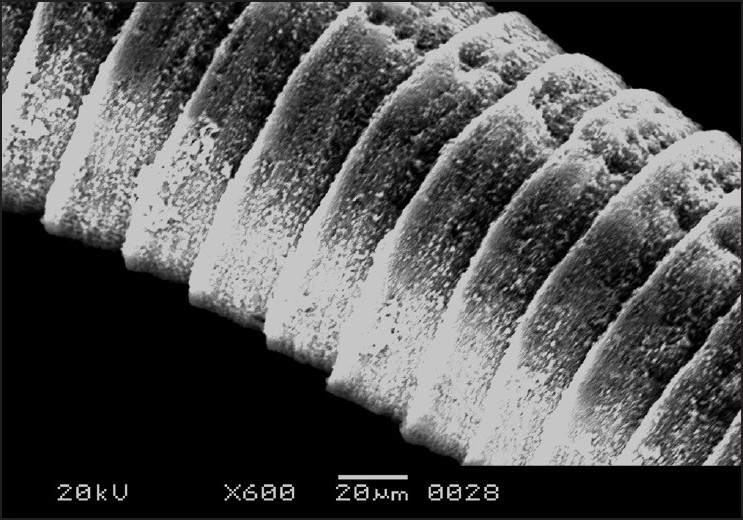
Scanning electron micrograph of untreated *R. echinobothrida* showing the body proper or the strobila, which consists of a chain of proglottides that are entirely covered with hair-like microtriches

The morphology of *R. echonobothrida* was recently described in detail.[11] In brief, the body is of a typical tapeworm, consisting of a knob-like scolex at the anterior end, followed by a flattened neck and an elongated body proper called strobila. On the scolex are four oval-shaped suckers that surround an apical opening called the rostellum [Figure 1]. Each sucker is an organ of attachment and possesses several rows of sharply pointed spines along its rim. The strobila is a highly elongated ribbon-like structure composed of a chain of conjoined segments called proglottides [Figure 2]. The entire body is covered with the tegument, which has hair-like filaments called microtriches throughout the surface, giving the overall surface a velvety appearance.

**Figure 3:**
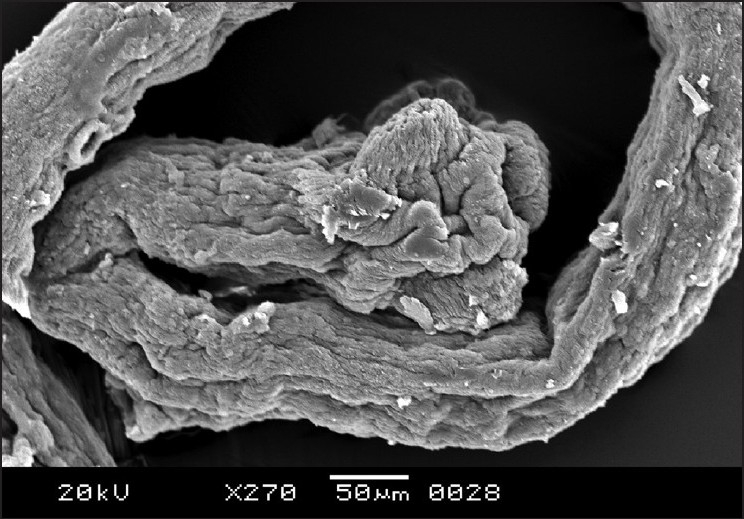
Scanning electron micrograph of *R. echinobothrida* treated with albendazole showing severe distortion of the entire body

**Figure 4:**
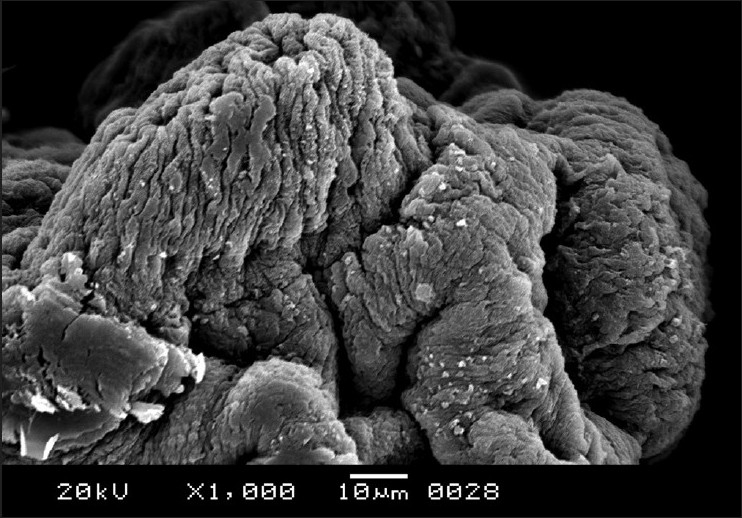
Scanning electron micrograph of *R. echinobothrida* treated with albendazole showing shrunken scolex

**Figure 5:**
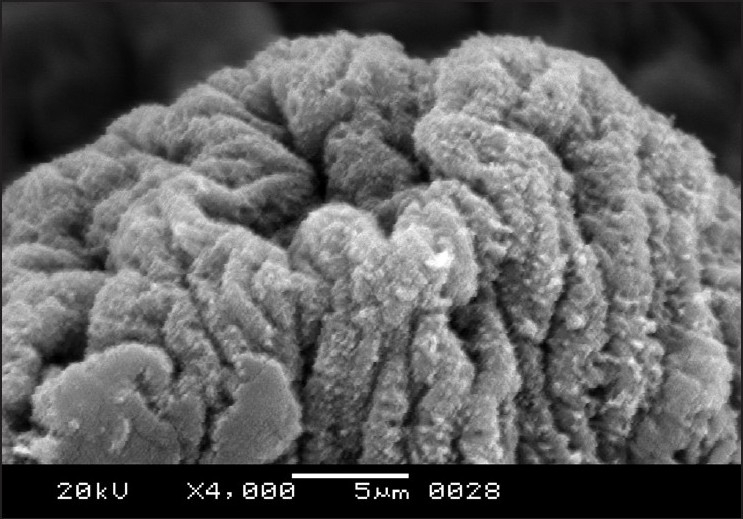
Scanning electron micrograph of *R. echinobothrida* treated with albendazole showing invaginated sucker

**Figure 6:**
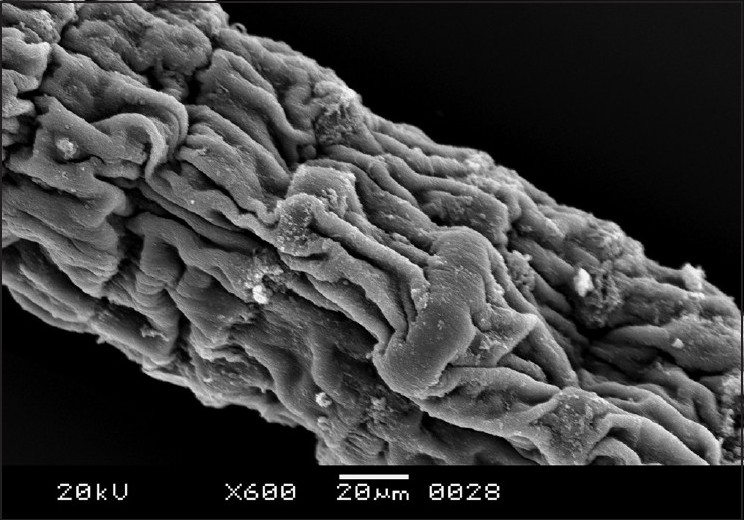
Scanning electron micrograph of *R. echinobothrida* treated with albendazole showing the deformed proglottides

**Figure 7:**
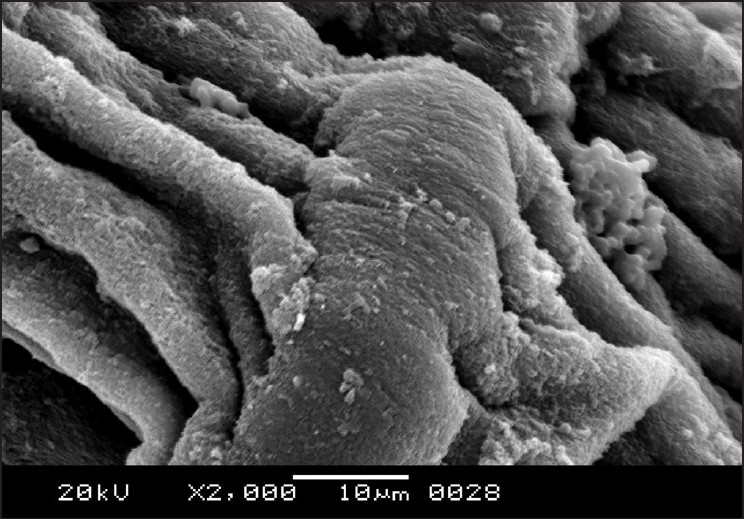
Scanning electron micrograph of *R. echinobothrida* treated with albendazole showing a highly constricted proglottid

**Figure 8:**
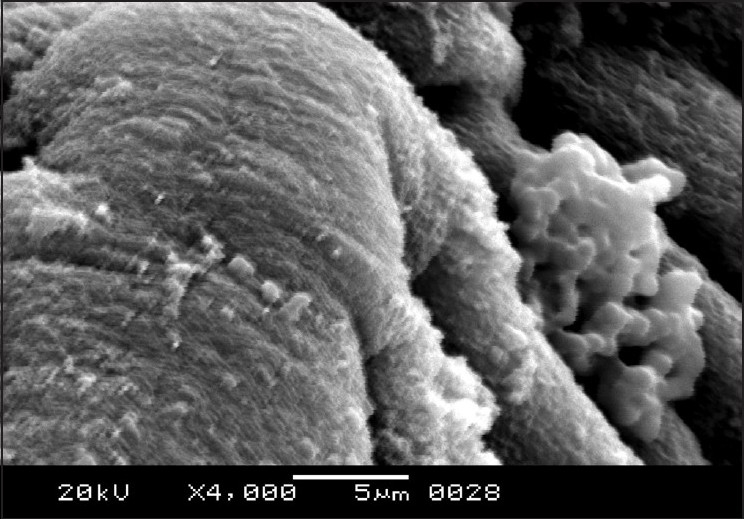
Scanning electron micrograph of *R. echinobothrida* treated with albendazole showing abnormal clumping on the surface of a proglottid

The cestodes treated with 20 mg/mL of the drug were chosen for scanning electron microscopy as the most obvious alterations of the morphology were shown at this concentration. The entire body was extensively deformed [Figure 3]. The scolex appeared greatly contracted, with layers of irregular tegumental folds [Figure 4]. The suckers were greatly contracted and invaginated [Figure 5]. The proglottides were also shrunken, and due to the severe shrinkage the chain-like body segments were lost [Figure 6]. The surface of the proglottides completely lost the smooth appearance [Figure 7]. Some malformed debris and clumping were visible [Figure 8].

## DISCUSSION

Albendazole and its related benzimidazoles are known to enter the helminths by passive diffusion through the external surface of helminths. These drugs directly cause disruption of the tegumental and muscle layers by binding specifically to β-tubulins, thereby inhibiting polymerization and functioning of the cellular motor proteins.[12,13] The tegument or cuticle is the fundamental interface of the helminth’s body with its environment and is responsible for selective absorption of nutrients, secretory activities, and sensory perception; this renders it specifically susceptible to anthelmintic agents. It has been frequently documented that the distinctive effect of anthelmintic drugs on cestodes is detrimental alterations and destruction of the tegument.[14,15]

Scanning electron microscopy has been the major tool in describing the direct effects of anthelmintic agents on cestodes. Formation of numerous blebs on the tegument which became detached, leaving debris only, rostellar disorganization and loss of the microtriches were observed for the effects of pure albendazole and its sulphoxide combination therapy on the human cestode, *Echinococcus granulosus*.[16] Treatment of *E. granulosus* and *Mesocestoides corti* with *a* combination of albendazole and praziquantel also resulted in the loss of sucker concavity, separation and disintegration of the germinal layers, loss of microtriches, and destruction of the tegument.[17,18] The damaging effects described for albendazole, flubendazole, and nitazoxanide are highly comparable and are typified by reductions in number and length of the microtriches, rostellar degeneration, formation of blebs on the tegument, loss of hooks and destruction of microtriches, and vesiculation in *E. granulosus*[19,20] and in *E. multiloculoris*.[21]

Several anthelmintic agents, including certain plant extracts, have been reportedly shown to cause considerable structural alterations on *R. echinobothrida*.[22] However, the nature of the changes is highly specific and different for each agent. Genistein and the extract of *Flemingia vestita* caused formation of tegumental cracks in the scolex, clumping of microtriches on the proglottides, and shrinkage of the tegument.[23] Stem bark extract of *Acacia caesia* was demonstrated to induce focal erosion and degeneration of the microtriches of the proglottides and distortion of suckers.[24] Root extract of *Millettia pachycarpa* also produced extensive truncation of the tegument, with formation of pits and vacuoles and massive erosion of the surface of the scolex.[25] The extensive shrinkage of the tegument and invagination of the suckers in the present study appear to be the characteristic effects of albendazole.

## CONCLUSIONS

The present observations clearly indicate that albendazole is highly effective against the cestode *R. echinobothrida*. The structural changes that were seen are unique in that such massive contraction of the tegument, extending throughout the entire length of the body, has never been described. Further, complete invagination of the suckers, formation of abnormal clumps on the proglottides, and distortion of microtriches are definitely the specific effects of the drug on cestodes. The result supports the proposition that albendazole should be a drug of choice against poultry cestodes.
